# Supplementary material for: Brain2GAN: Feature-disentangled neural encoding and decoding of visual perception in the primate brain
Source: PLoS Comput Biol. 2024 May 6;20(5):e1012058. doi: 10.1371/journal.pcbi.1012058 (PMC11098503; doi:10.1371/journal.pcbi.1012058)
Supplement: S1 Appendix — Fig A: Encoding performance. The effectiveness of each encoding model is assessed using the Pearson correlation coefficients between predicted and recorded neural responses. The first and second graphs denote discriminative and generative representations, respectively. Fig B: Generative-based encoding performance. For each individual microelectrode unit, we fit three encoding models based on three distinct feature representations: z-, w− and CLIP-latent representations. As such, we fit 3× 1024 independent encoders, resulting in 3× 1024 predicted neural responses. The scatterplots display the prediction-target correlation (r) of one encoding model on the X-axis and another encoding model on the Y-axis to investigate the relationship between the two. Each dot represents the performance of one modeled microelectrode unit in terms of both encoding models (so, 1024 dots per plot). The diagonal represents equal performance between both models. It is clear to see that w-latents always outperform z- and CLIP-latents because most dots lie in the direction of the w-axis (above the diagonal). Fig C: Qualitative results. This figure shows the 100 test set stimuli (top row) and their reconstructions from brain activity from subject 1 (middle row) and subject 2 (bottom row). (PDF) [file pcbi.1012058.s001.pdf]

S1 Appendix: Results For Macaque #2

We repeated the passive fixation experiment using brain responses from V1, V2, V3 and V4 in a second macaque (male, 9 years old) with silicone-based electrodes. Note that this subject has no electrode arrays implanted in higher-level visual areas. All set-up and collection procedures, including recording equipment and the preprocessing, were identical to those of the first macaque. The encoding performance was quantified as the Pearson product-moment correlation coefficient between the predicted and recorded responses (Figs A and B in S1 Appendix). Among the generative-based encoders, the w-latent-based encoder significantly outperformed the z-based encoder (2-Sample T-Test; $t(2046) = -8.3180$, $p = 1.6\text{e}{-16}$) but not the CLIP-based encoder (2-Sample T-Test; $t(2046) = -0.7301$, $p = 0.4653$). In addition, CLIP-based encoding also outperformed z-based encoding (2-Sample T-Test; $t(2046) = 7.6463$, $p = 3.16\text{e}{-14}$).

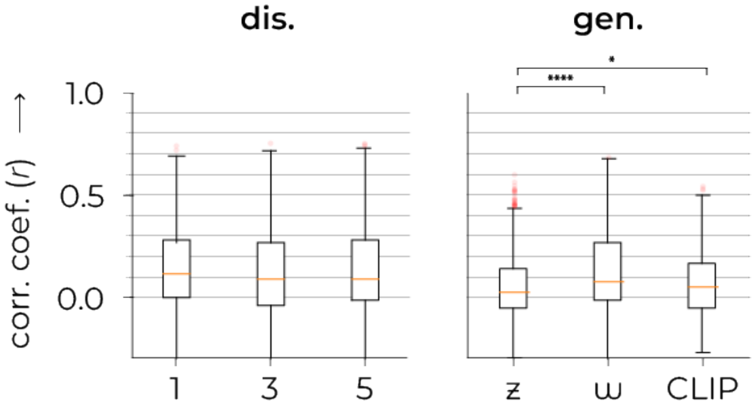

Figure A: **Encoding performance.** The effectiveness of each encoding model is assessed using the Pearson correlation coefficients between predicted and recorded neural responses. The first and second graphs denote discriminative and generative representations, respectively.

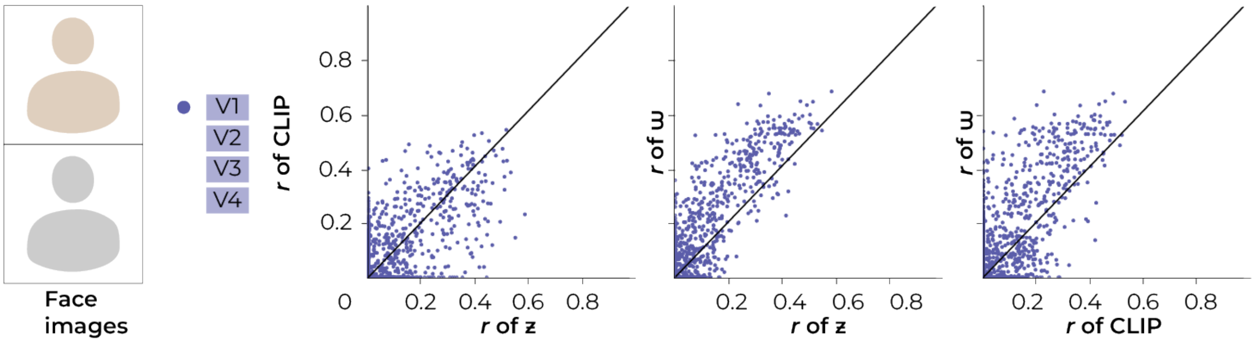

Figure B: **Generative-based encoding performance.** For each individual microelectrode unit, we fit three encoding models based on three distinct feature representations: z-, $w-$ and CLIP-latent representations. As such, we fit $3\times$ 1020 independent encoders, resulting in $3\times$ 1024 predicted neural responses. The scatterplots display the prediction-target correlation (r) of one encoding model on the X-axis and another encoding model on the Y-axis to investigate the relationship between the two. Each dot represents the performance of one modeled microelectrode unit in terms of both encoding models (so, 1024 dots per plot). The diagonal represents equal performance between both models. It is clear to see that w-latents always outperform z- and CLIP-latents because most dots lie in the direction of the w-axis (above the diagonal). Face images in this figure are replaced for copyright reasons. The original version of the figure can be accessed here.

The reconstruction from areas V1, V2, V3 and V4 in the second macaque (Fig C in S1 Appendix, bottom row) were highly similar to the perceived stimuli but contained consistently less intricate detail compared to the reconstructions from areas V1, V4 and IT in the first macaque (Fig C in S1 Appendix, middle row - for reference).

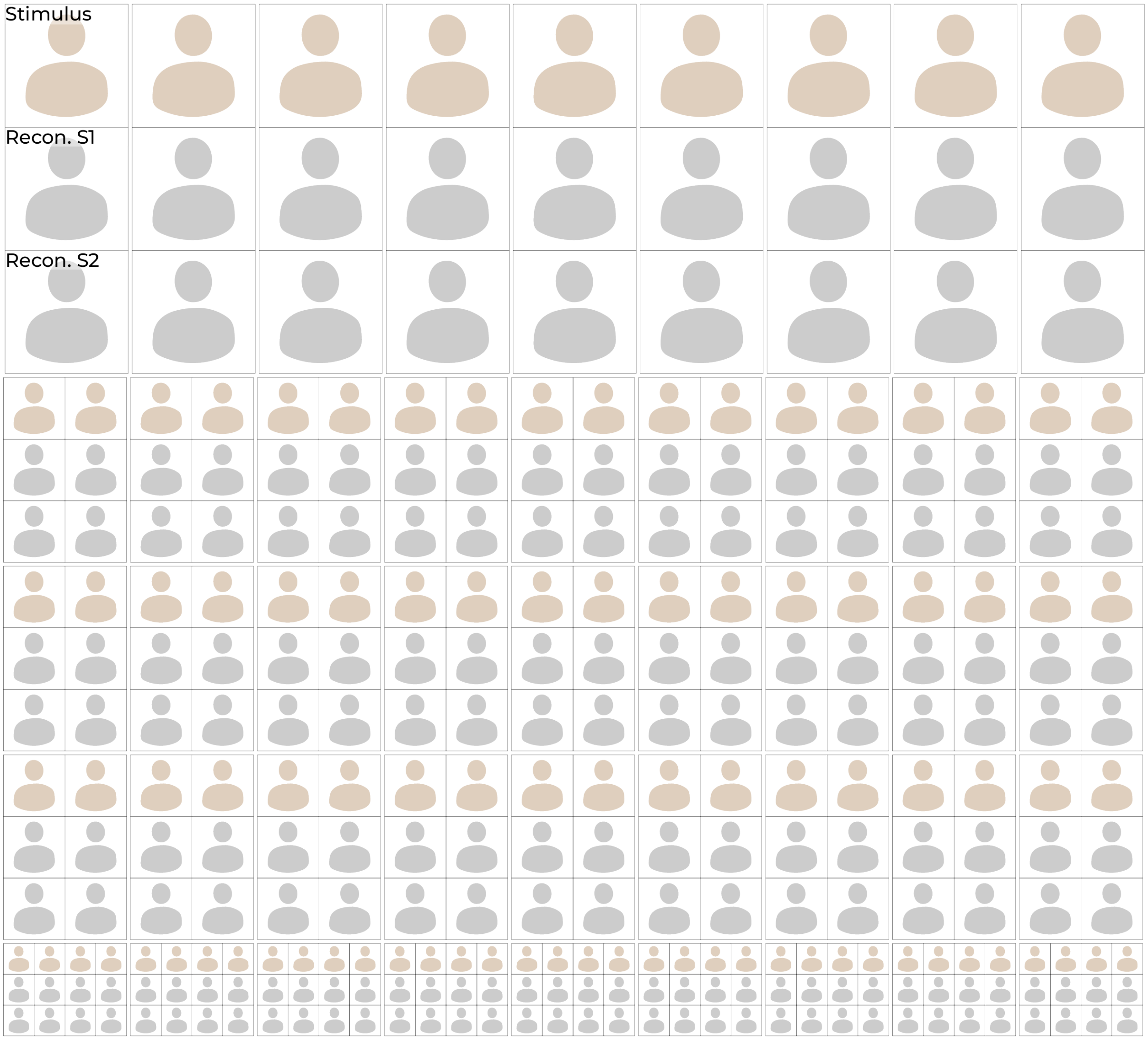

Figure C: **Qualitative results.** This figure shows the 100 test set stimuli (top row) and their reconstructions from brain activity in V1, V4 and IT from subject 1 (middle row) and subject 2 (bottom row). Face images in this figure are replaced for copyright reasons. The original version of the figure can be accessed here.
