# Supplementary material for: Brain2GAN: Feature-disentangled neural encoding and decoding of visual perception in the primate brain
Source: PLoS Comput Biol. 2024 May 6;20(5):e1012058. doi: 10.1371/journal.pcbi.1012058 (PMC11098503; doi:10.1371/journal.pcbi.1012058)
Supplement: S2 Appendix — Fig A: Qualitative results for face images. Test set stimuli (top), ‘original’ reconstructions from brain activity via w-latents (middle) and reconstructions from brain activity via z-latents. Fig B: Qualitative results for natural images. Test set stimuli (top), ‘original’ reconstructions from brain activity via w-latents (middle) and reconstructions from brain activity via z-latents. (PDF) [file pcbi.1012058.s002.pdf]

S2 Appendix: Reconstruction via z-Latents

The reconstructions from z-latents not only demonstrate superior performance using w-latents in *conditional* image generation (Fig A in S2 Appendix) but also that this disentanglement enables *unconditional* image generation using GANs (Fig B in S2 Appendix).

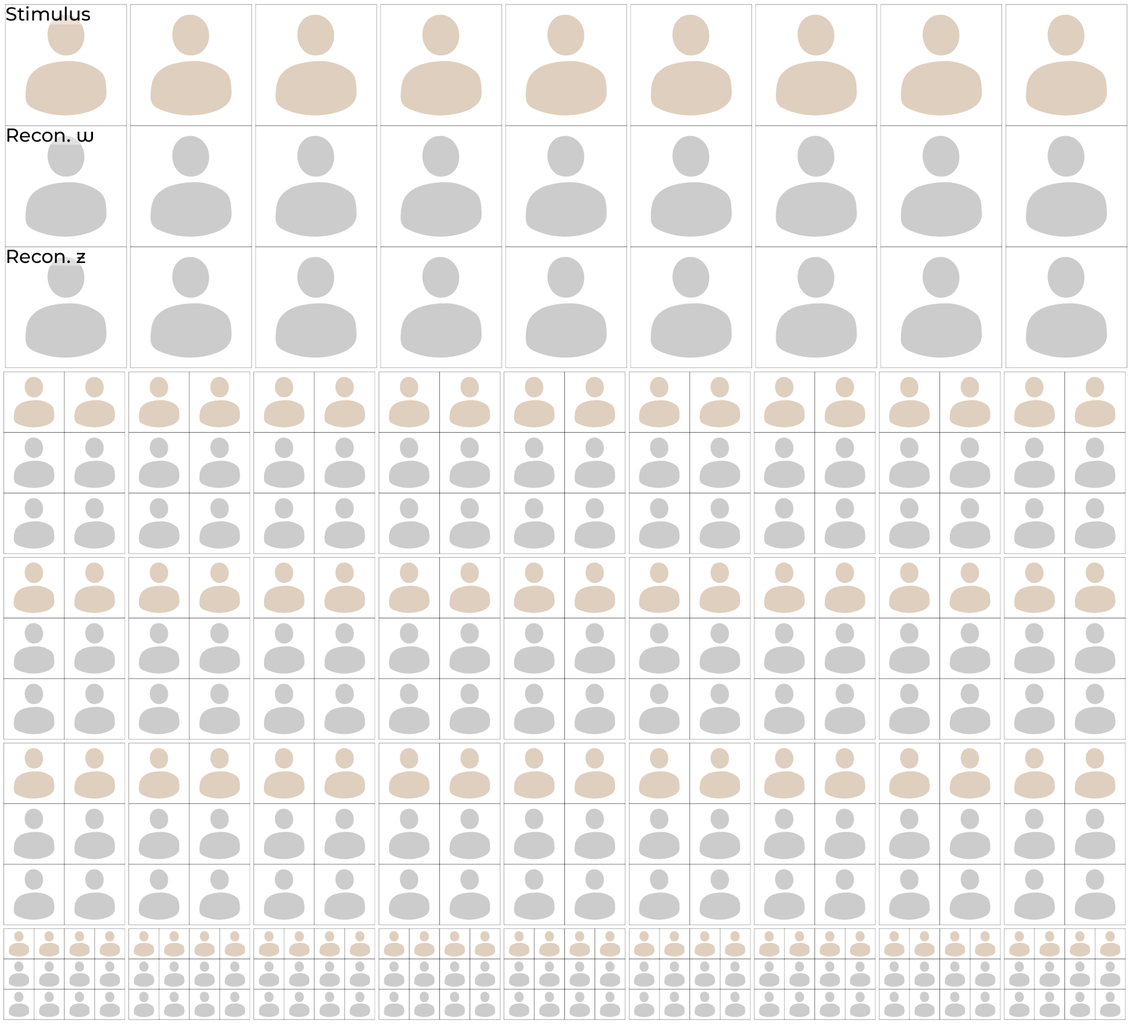

Figure A: **Qualitative results for face images.** Test set stimuli (top), 'original' reconstructions from brain activity in V1, V4 and IT via w-latents (middle) and reconstructions from brain activity in V1, V4 and IT via z-latents. Face images in this figure are replaced for copyright reasons. The original version of the figure can be accessed here.

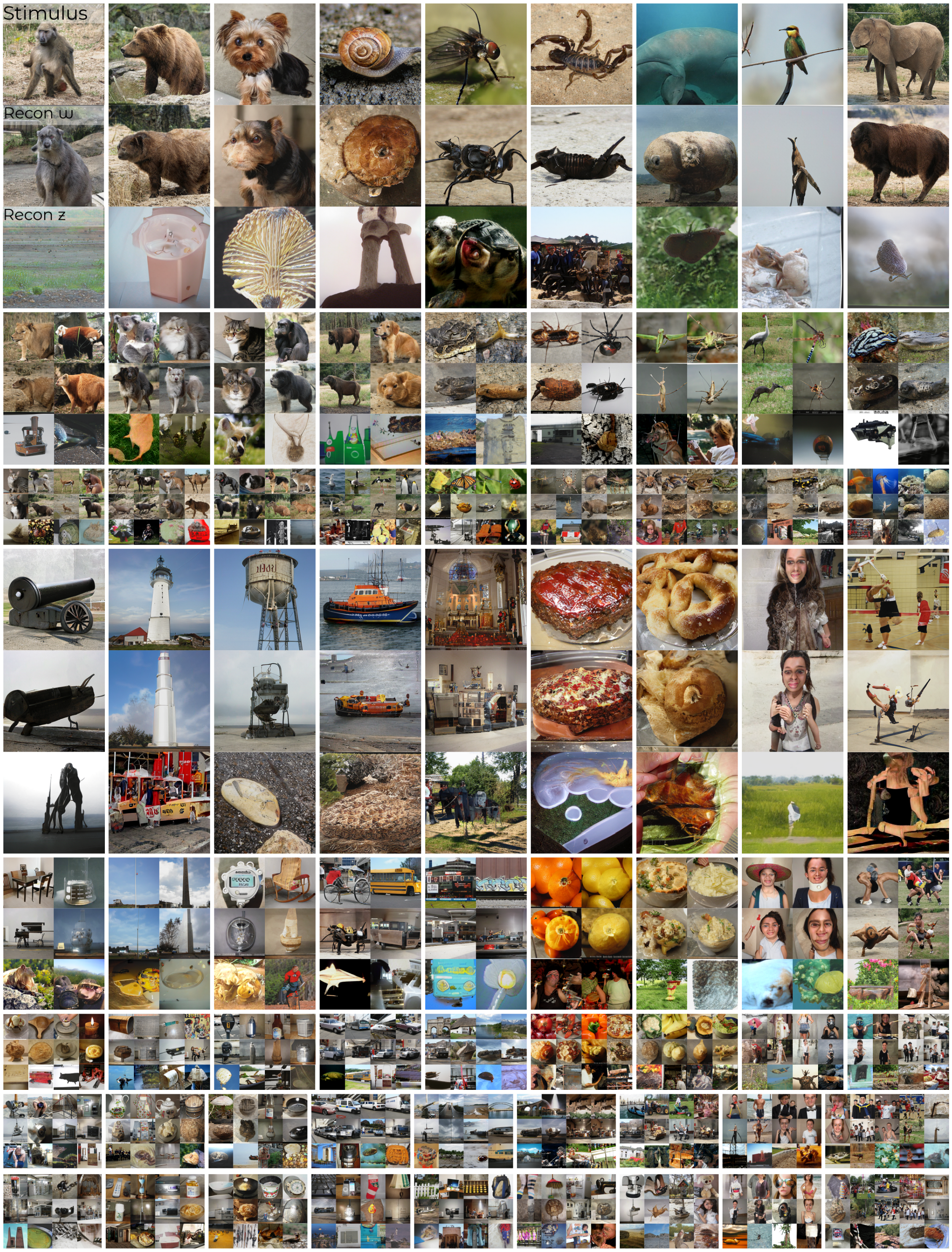

Figure B: **Qualitative results for natural images.** Test set stimuli (top), 'original' reconstructions from brain activity in V1, V4 and IT via w-latents (middle) and reconstructions from brain activity in V1, V4 and IT via z-latents.
