# Supplementary material for: Brain2GAN: Feature-disentangled neural encoding and decoding of visual perception in the primate brain
Source: PLoS Comput Biol. 2024 May 6;20(5):e1012058. doi: 10.1371/journal.pcbi.1012058 (PMC11098503; doi:10.1371/journal.pcbi.1012058)
Supplement: S3 Appendix — Table A: Quantitative results. Reconstruction performance (mean ± std.error) in terms of six metrics of perceptual cosine similarity using the five MaxPool layer outputs of VGG16 for face or image recognition and latent cosine similarity between w-latents of stimuli and their reconstructions when using the recordings from all recording sites (i.e., V1, V4 and IT together). The first row shows the original reconstruction performance from the manuscript, and the second and third rows of the baseline using the prior of 10,000 and 6,000,000 images, respectively. Fig A: Qualitative results for face images (prior = 10,000). Test set stimuli (top), ‘original’ reconstructions from brain activity using linear decoding (middle) and reconstructions from brain activity using the baseline approach. Fig B: Qualitative results for face images (prior = 6,000,000). Test set stimuli (top), ‘original’ reconstructions from brain activity using linear decoding (middle) and reconstructions from brain activity using the baseline approach. Fig C: Qualitative results for natural images (prior = 10,000). Test set stimuli (top), ‘original’ reconstructions from brain activity using linear decoding (middle) and reconstructions from brain activity using the baseline approach. Fig D: Qualitative results for natural images (prior = 60,000,000). Test set stimuli (top), ‘original’ reconstructions from brain activity using linear decoding (middle) and reconstructions from brain activity using the baseline approach. (PDF) [file pcbi.1012058.s003.pdf]

S3 Appendix: Reconstruction Baseline

We implemented a reconstruction method based on the work of [28] which estimated the posterior probability of a stimulus given the target responses, considering both the likelihood and prior probability of that stimulus. We modified this approach to incorporate the advantages of generative modeling, thereby aligning it with our central theme and enhancing reconstruction quality. In brief, we sampled a large number ($N = 10,000$ and $N = 6,000,000$ [26] of w-latent vectors and fed them to our encoder to predict neural responses. For each test set example, we selected a subset ($n = 100$) of responses that were most similar to the observed test set response and averaged their corresponding w-latents. This averaged latent was fed to the generator for reconstruction. The qualitative results for faces can be found in Figs A and B in S3 Appendix, and for natural images in Figs C and D in S3 Appendix. Quantitative results can be found in Table A in S3 Appendix.

Perceptually, it is easy to see that our original reconstructions outperformed those by the baseline. To our surprise, we also observed that the reconstructions using the larger prior of 6 million images were perceptually inferior compared to those using the smaller prior of 10,000 images, despite selecting what are assumed to be the best hundred latents based on their predicted similarity to the brain responses. The performance discrepancy between our decoding method and the baseline could be due to the fundamental differences in how each method relates brain activity to latents. That is, our reconstruction method utilizes a multivariate approach from all responses to predict each latent dimension independently (512 in total) such that the combined effect of all responses is captured per latent dimension. In turn, the baseline method employs the encoding model which is a mass univariate approach that predicts single neural responses (960 in total) from all latent dimensions. The encoding performance may not directly correlate with the reconstruction performance because the encoding model does not consider how all neural responses jointly relate to each latent dimension. As such, this could result in the selection of latents that are suboptimal for the purpose of visual reconstruction, even if they are good at predicting individual neural responses.

Table A: **Quantitative results.** Reconstruction performance ($mean \pm std.error$) in terms of six metrics of perceptual cosine similarity using the five MaxPool layer outputs of VGG16 for face or image recognition and latent cosine similarity between w-latents of stimuli and their reconstructions when using the recordings from all recording sites (i.e., V1, V4 and IT together). The first row shows the original reconstruction performance from the manuscript, and the second and third rows of the baseline using the prior of 10,000 and 6,000,000 images, respectively.

		**VGG16-1 sim.**	**VGG16-2 sim.**	**VGG16-3 sim.**	**VGG16-4 sim.**	**VGG16-5 sim.**	**Lat. sim.**
Face images	orig.	0.7871 ± 0.0102	0.7681 ± 0.0075	0.5874 ± 0.0075	0.6170 ± 0.0085	0.5940 ± 0.0104	0.5548 ± 0.0045
	10k	0.6380 ± 0.0133	0.6712 ± 0.0059	0.4628 ± 0.0059	0.4702 ± 0.0080	0.4241 ± 0.0104	0.4762 ± 0.0055
	6M	0.5873 ± 0.0118	0.6214 ± 0.0048	0.3961 ± 0.0048	0.3599 ± 0.0082	0.3370 ± 0.0131	0.4068 ± 0.0071
Natural images	orig.	0.4083 ± 0.0036	0.3322 ± 0.0036	0.2555 ± 0.0025	0.2192 ± 0.0043	0.2497 ± 0.0066	0.8032 ± 0.0032
	10k	0.3723 ± 0.0050	0.3050 ± 0.0026	0.2267 ± 0.0026	0.1636 ± 0.0039	0.1438 ± 0.0061	0.7563 ± 0.0049
	6M	0.3730 ± 0.0050	0.3041 ± 0.0024	0.2218 ± 0.0024	0.1545 ± 0.0031	0.1341 ± 0.0057	0.5963 ± 0.0099

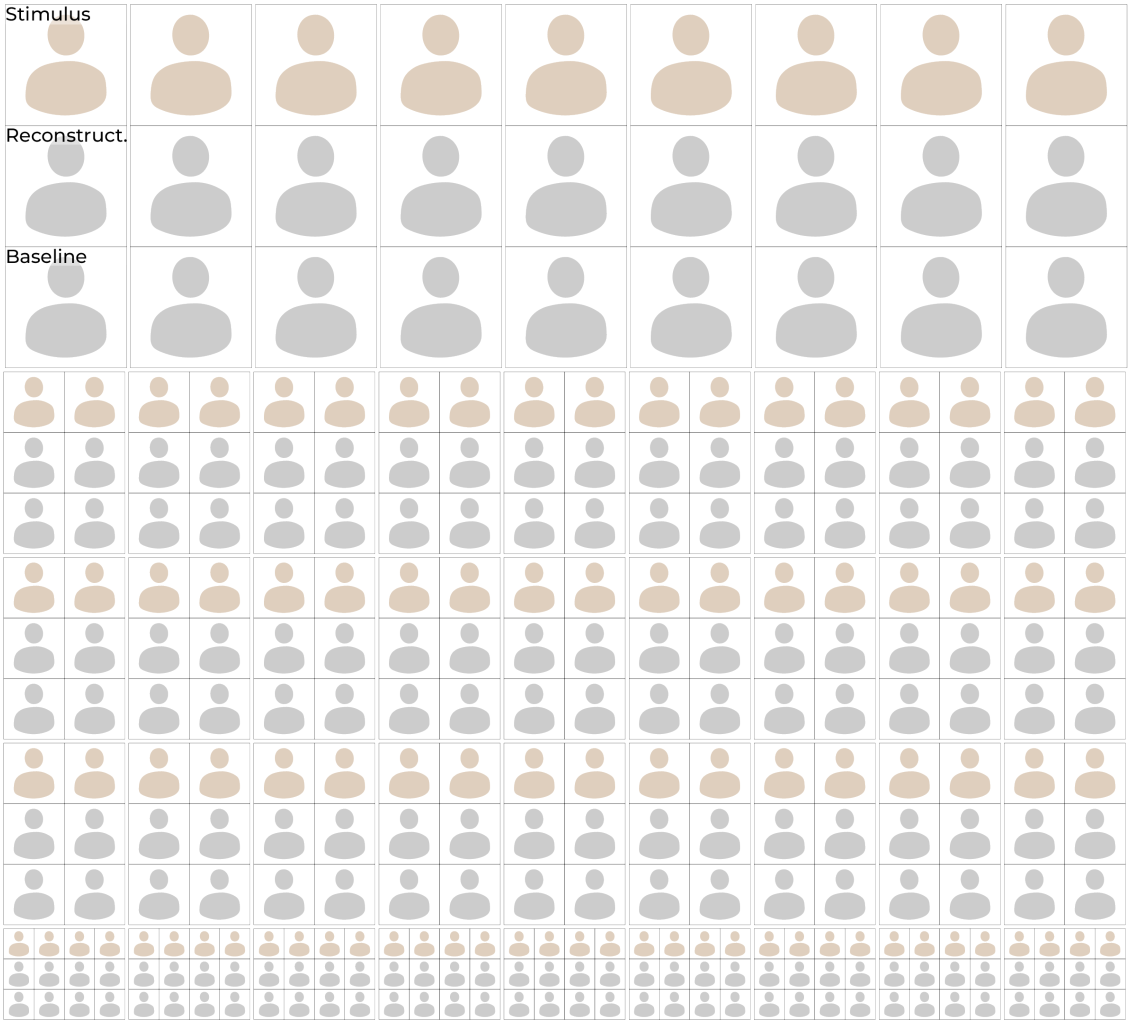

Figure B: **Qualitative results for face images (prior=10,000).** Test set stimuli (top), 'original' reconstructions from brain activity in V1, V4 and IT using linear decoding (middle) and reconstructions from brain activity in V1, V4 and IT using the baseline approach. Face images in this figure are replaced for copyright reasons. The original version of the figure can be accessed here.

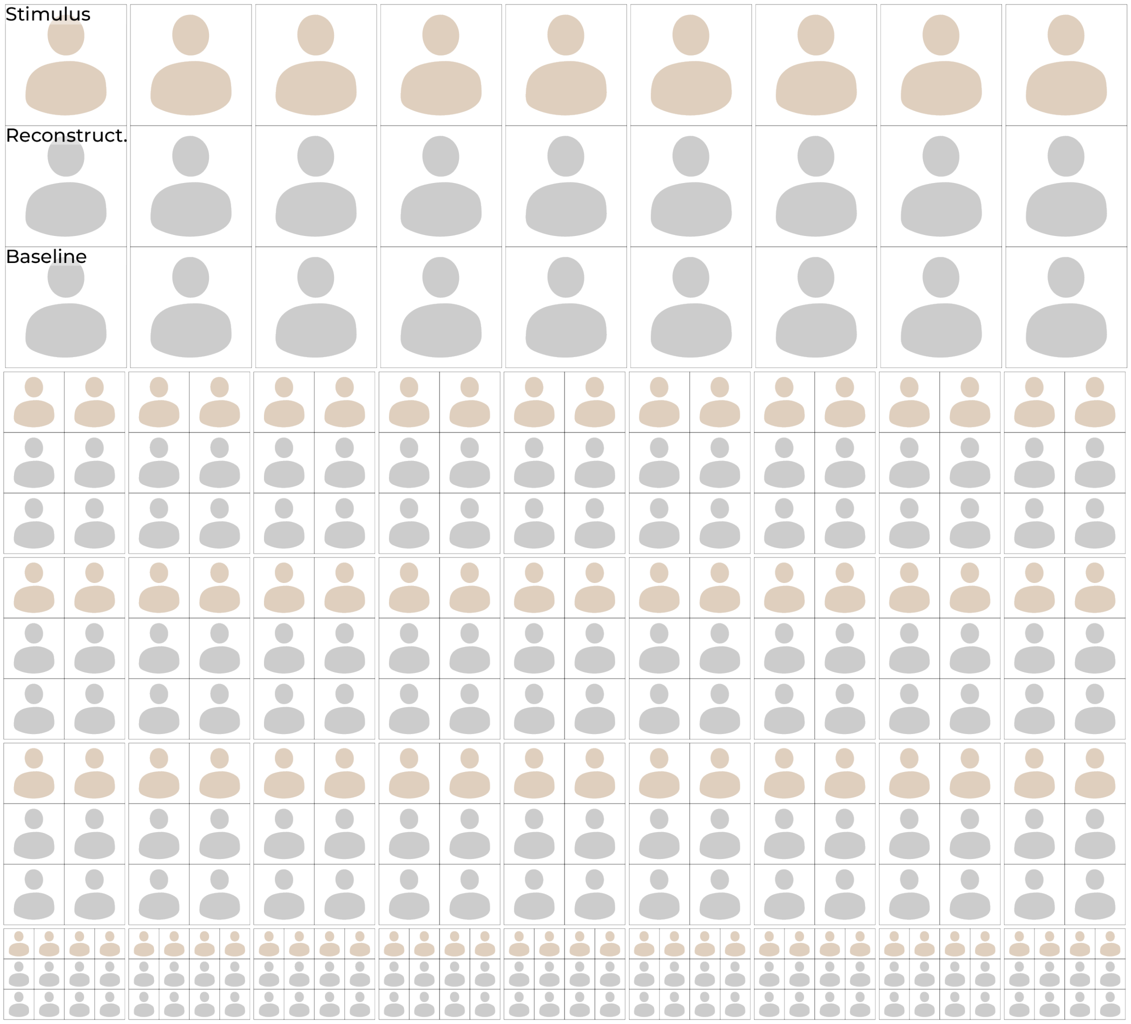

Figure C: **Qualitative results for face images (prior=6,000,000).** Test set stimuli (top), 'original' reconstructions from brain activity in V1, V4 and IT using linear decoding (middle) and reconstructions from brain activity in V1, V4 and IT using the baseline approach. Face images in this figure are replaced for copyright reasons. The original version of the figure can be accessed here.

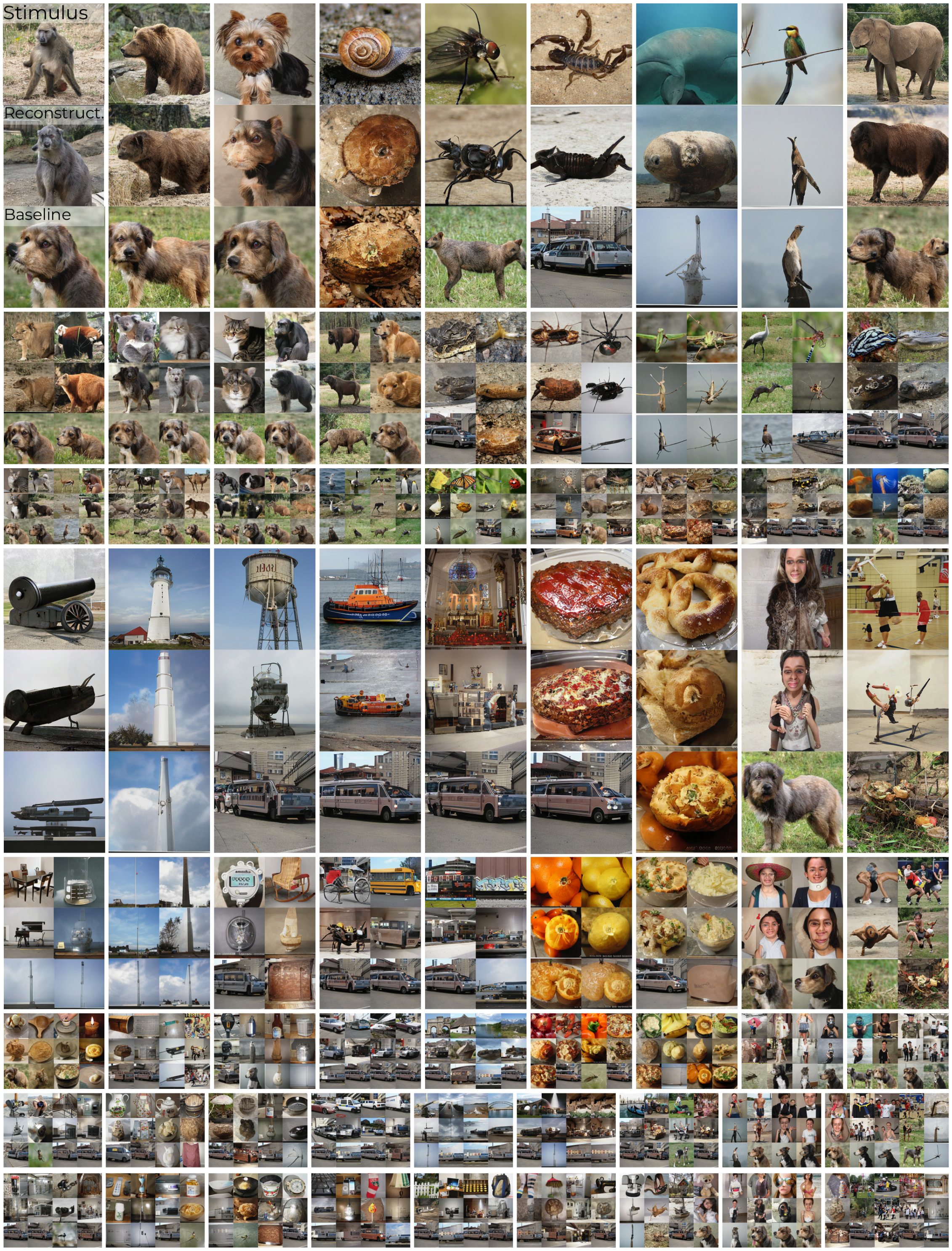

Figure D: **Qualitative results for natural images (prior=10,000).** Test set stimuli (top), 'original' reconstructions from brain activity in V1, V4 and IT using linear decoding (middle) and reconstructions from brain activity in V1, V4 and IT using the baseline approach.

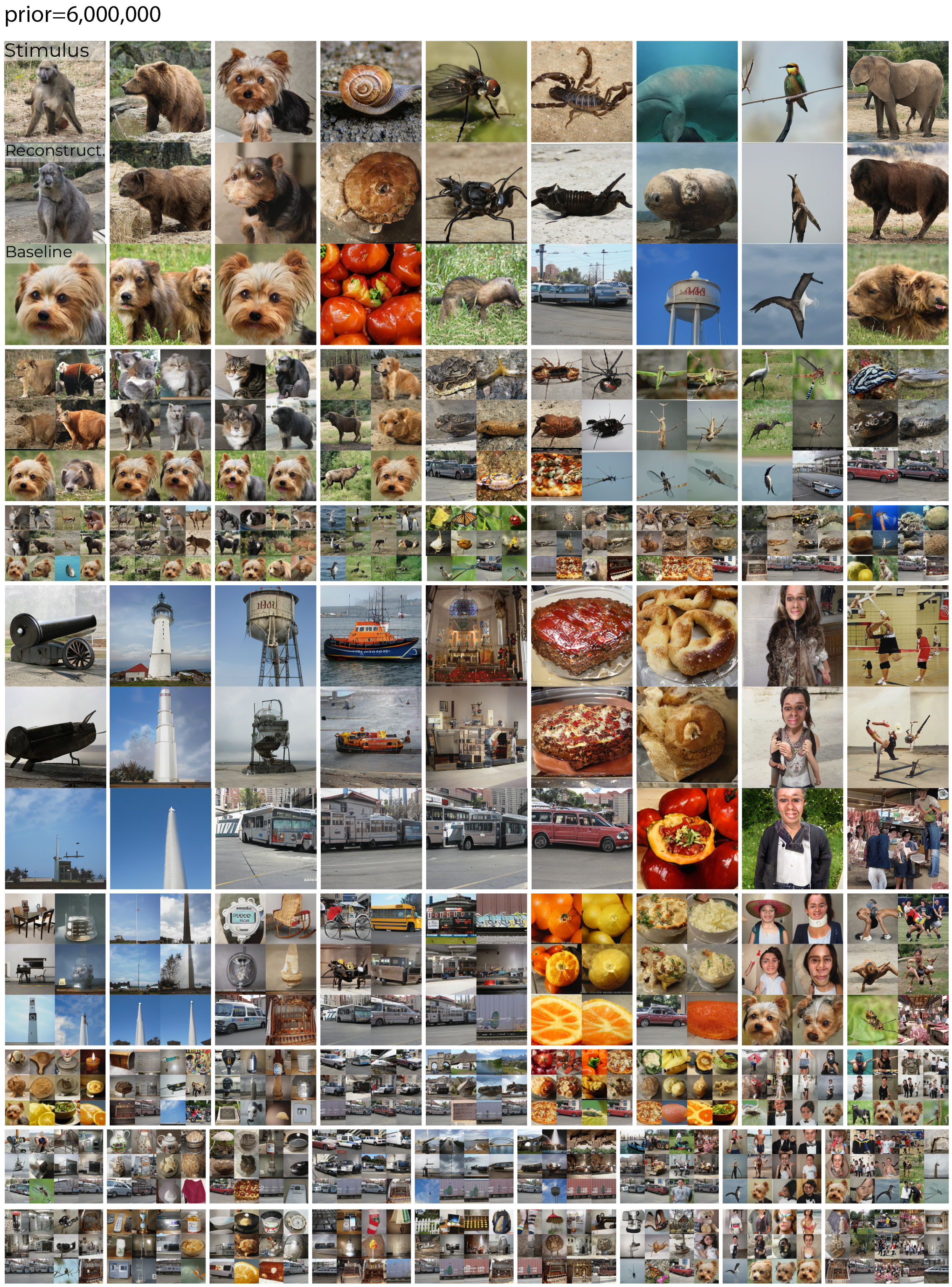

Figure E: **Qualitative results for natural images (prior=6,000,000).** Test set stimuli (top), 'original' reconstructions from brain activity in V1, V4 and IT using linear decoding (middle) and reconstructions from brain activity in V1, V4 and IT using the baseline approach.
