# Supplementary material for: Brain2GAN: Feature-disentangled neural encoding and decoding of visual perception in the primate brain
Source: PLoS Comput Biol. 2024 May 6;20(5):e1012058. doi: 10.1371/journal.pcbi.1012058 (PMC11098503; doi:10.1371/journal.pcbi.1012058)
Supplement: S4 Appendix — Table A: Quantitative results. Reconstruction performance (mean ± std.error) in terms of six metrics of perceptual cosine similarity using the five MaxPool layer outputs of VGG16 for object recognition and latent cosine similarity between w-latents of stimuli and their reconstructions when using the recordings from all recording sites (i.e., V1, V4 and IT together). The first row shows the original reconstruction performance from the manuscript and the second row of the leave-one-example-out analysis. Fig A: Qualitative reconstruction results: training examples that are used for testing (top) row and their reconstructions from brain activity (bottom row) via w-latents. (PDF) [file pcbi.1012058.s004.pdf]

S4 Appendix: Leave-One-Example-Out Analysis

In our study, we used the average w-latent of each class for the test set since it guaranteed high image quality and because variation was not required as only one image per category was needed. The average w-latent lives in the same w-space as the other w-latents, so there should be no advantage during reconstruction. To validate that our results were not a confound of using the average w-latent, we conducted an additional analysis where we trained a decoder on the first 19 training examples of each class and tested it on the remaining training example. We found that the reconstructions still resembled the stimuli, although there was a noticeable decrease in quality. This was attributed to the lower signal-to-noise ratio resulting from using a single training response. Some stimulus-reconstruction examples are presented in panel A of Fig A in S4 Appendix. For reference, panel B of Fig A in S4 Appendix shows the reconstructions based on the "original" test set. These were obtained using all training examples but decoded from a single response in the test set. This approach was chosen to match the signal-to-noise ratio conditions of the aforementioned leave-one-example-out analysis. Furthermore, Table A in S4 Appendix details the performance metrics of these leave-one-example-out reconstructions in comparison to the 'original' reconstruction performance based on the use of single test responses.

Table A: **Quantitative results.** Reconstruction performance in terms of six metrics of perceptual cosine similarity using the five MaxPool layer outputs of VGG16 for object recognition and latent cosine similarity between w-latents of stimuli and their reconstructions ($mean \pm std.error$) when using the recordings from all recording sites (i.e., V1, V4 and IT together). The first row shows the original reconstruction performance from the manuscript and the second row of the leave-one-example-out analysis.

	**VGG16-1 sim.**	**VGG16-2 sim.**	**VGG16-3 sim.**	**VGG16-4 sim.**	**VGG16-5 sim.**	**Lat. sim.**
orig. (single)	0.3923 ± 0.0048	0.3205 ± 0.0022	0.2409 ± 0.0022	0.1870 ± 0.0033	0.1963 ± 0.0056	0.7013 ± 0.0044
l-o-e-o.	0.3771 ± 0.0047	0.3108 ± 0.0022	0.2317 ± 0.0022	0.1696 ± 0.0028	0.1634 ± 0.0048	0.7013 ± 0.0044

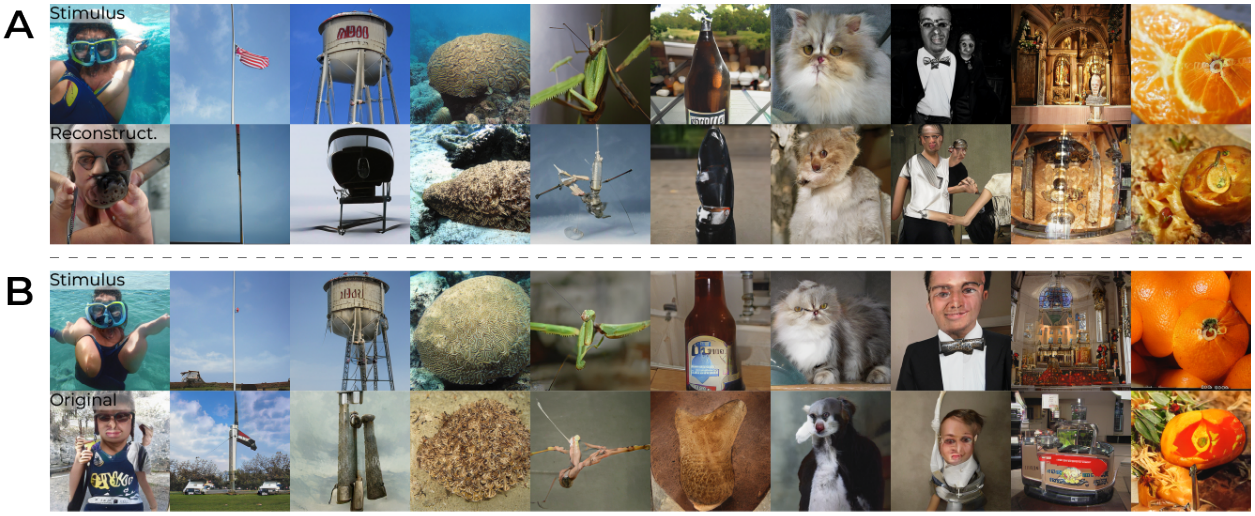

Figure A: **Qualitative reconstruction results.** Training examples that are used for testing (top) row and their reconstructions from brain activity in V1, V4 and IT (bottom row) via w-latents.
