# Supplementary material for: Brain2GAN: Feature-disentangled neural encoding and decoding of visual perception in the primate brain
Source: PLoS Comput Biol. 2024 May 6;20(5):e1012058. doi: 10.1371/journal.pcbi.1012058 (PMC11098503; doi:10.1371/journal.pcbi.1012058)
Supplement: S5 Appendix — Table A: Quantitative results. Reconstruction performance (mean ± std.error) in terms of six metrics of perceptual cosine similarity using the five MaxPool layer outputs of VGG16 for object recognition and latent cosine similarity between w-latents of stimuli and their reconstructions when using the recordings from all recording sites (i.e., V1, V4 and IT together). The first row shows the original reconstruction performance from the manuscript and the second row of the leave-one-class-out analysis. Fig A: Qualitative reconstruction results: test set stimuli (top) and their reconstructions from brain activity when the training examples of their class are excluded from training (middle). The original reconstructions, when all classes are included during training, are also displayed for reference. (PDF) [file pcbi.1012058.s005.pdf]

S5 Appendix: Leave-One-Class-Out Analysis

For each target category, we fit a decoder on the 199 other categories $\times$ 20 training examples, after which we predicted and reconstructed the test stimulus of the remaining class that was not included during training. We found these reconstructions to be highly consistent with the original reconstructions when all classes were included during training (Fig A and Table A in S5 Appendix). This finding strongly supports the assertion that our model's focus extends beyond mere classification.

Table A: **Quantitative results.** Reconstruction performance ($mean \pm std.error$) in terms of six metrics of perceptual cosine similarity using the five MaxPool layer outputs of VGG16 for object recognition and latent cosine similarity between w-latents of stimuli and their reconstructions when using the recordings from all recording sites (i.e., V1, V4 and IT together). The first row shows the original reconstruction performance from the manuscript and the second row of the leave-one-class-out analysis.

	**VGG16-1 sim.**	**VGG16-2 sim.**	**VGG16-3 sim.**	**VGG16-4 sim.**	**VGG16-5 sim.**	**Lat. sim.**
orig.	0.4083 ± 0.0036	0.3322 ± 0.0036	0.2555 ± 0.0025	0.2192 ± 0.0043	0.2497 ± 0.0066	0.8032 ± 0.0032
l-o-c-o.	0.4039 ± 0.0052	0.3287 ± 0.0024	0.2500 ± 0.0024	0.2049 ± 0.0038	0.2185 ± 0.0056	0.7622 ± 0.0037

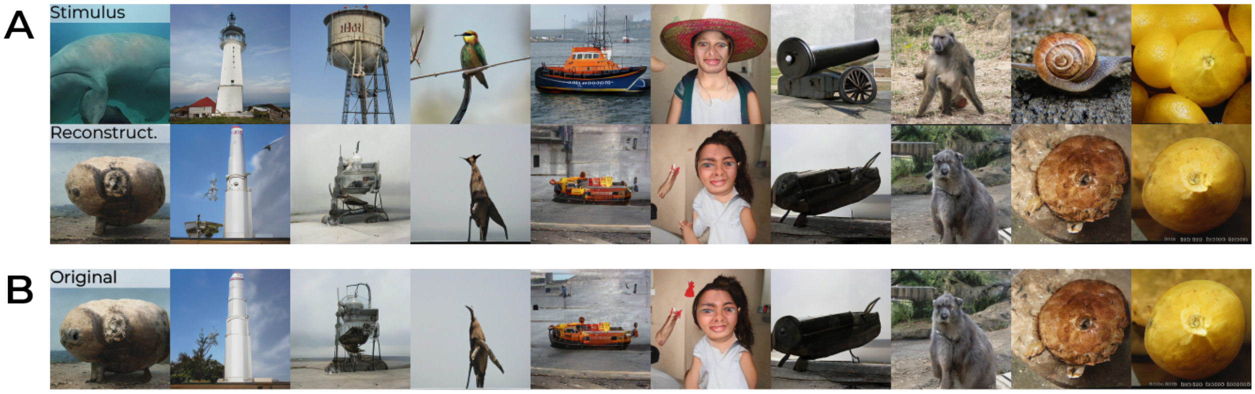

Figure A: **Qualitative reconstruction results.** Test set stimuli (top) and their reconstructions from brain activity in V1, V4 and IT when the training examples of their class are excluded from training (middle). The original reconstructions, when all classes are included during training, are also displayed for reference.
