# Supplementary figures and images for: Brain2GAN: Feature-disentangled neural encoding and decoding of visual perception in the primate brain
Source: PLoS Comput Biol. 2024 May 6;20(5):e1012058. doi: 10.1371/journal.pcbi.1012058 (PMC11098503; doi:10.1371/journal.pcbi.1012058)

## S6 Appendix: Permutation Test Analysis

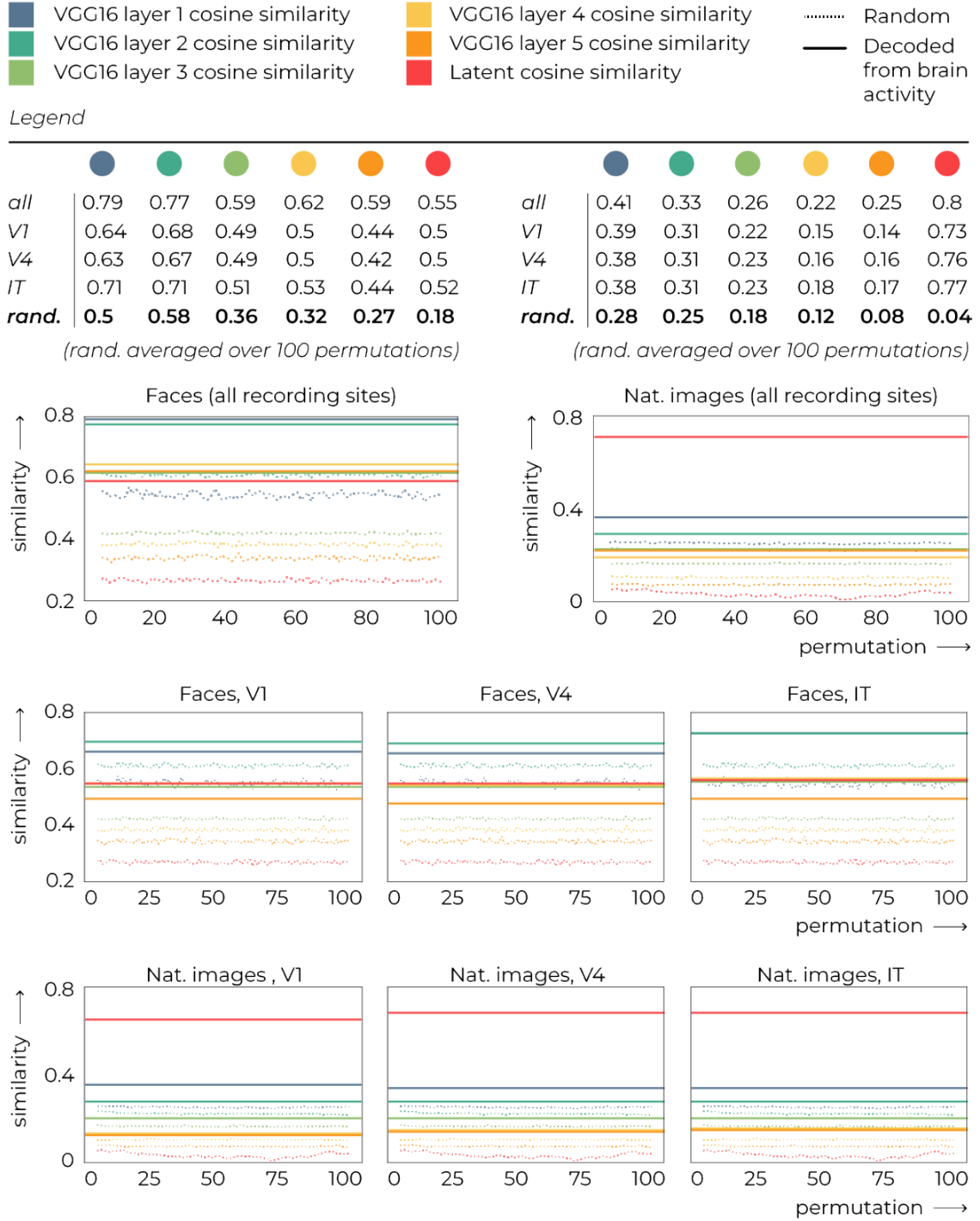

Supplement: S6 Appendix — Fig A: Permutation results. The quantitative results were verified with a permutation test as follows: per iteration, 100 and 200 latents (and their corresponding images) were randomly sampled for faces and natural images, respectively, to evaluate their similarity to the stimuli in terms of the six similarity metrics. In the above graphs, these similarity metrics were plotted over 100 iterations and we discovered that random samples were never better than our predictions from brain activity. (PDF) [file pcbi.1012058.s006.pdf]
