# Supplementary material for: Brain2GAN: Feature-disentangled neural encoding and decoding of visual perception in the primate brain
Source: PLoS Comput Biol. 2024 May 6;20(5):e1012058. doi: 10.1371/journal.pcbi.1012058 (PMC11098503; doi:10.1371/journal.pcbi.1012058)
Supplement: S7 Appendix — Fig A: Visual guide. For the six similarity metrics, we display the five lowest and highest stimulus-reconstruction pairs from the datasets of faces (left panel) and natural images (right panel). The top row denotes the stimulus and the bottom row the reconstruction from brain activity. Face images in this figure are replaced for copyright reasons. The original version of the figure can be accessed here. (PDF) [file pcbi.1012058.s007.pdf]

S7 Appendix: Visual Guide

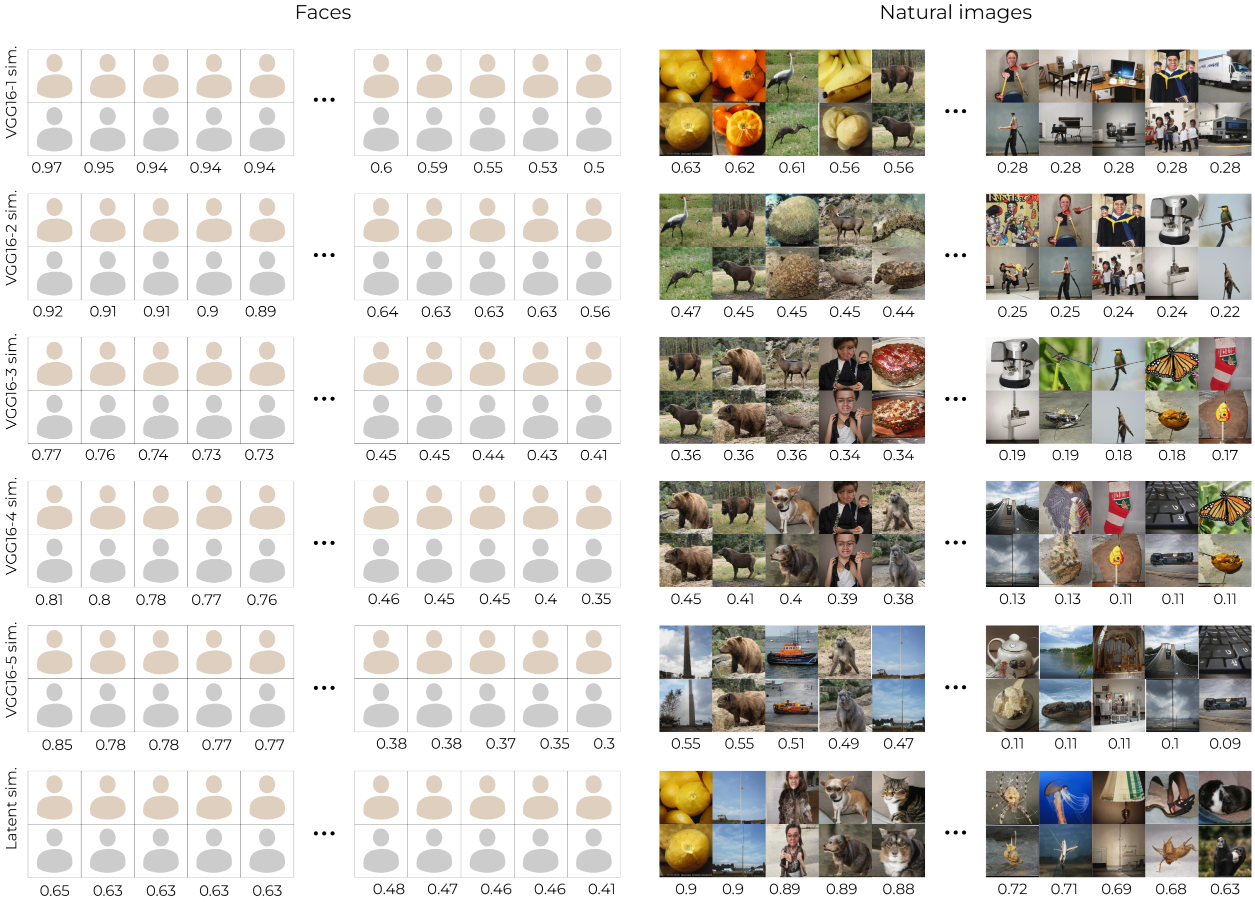

Fig A: **Visual guide.** For the six similarity metrics, we display the five lowest and highest stimulus-reconstruction pairs from the dataset of faces- (left) and natural images dataset (right). The top row denotes the stimulus and the bottom row the reconstruction from brain activity. Face images in this figure are replaced for copyright reasons. The original version of the figure can be accessed here.
