# Supplementary material for: Brain2GAN: Feature-disentangled neural encoding and decoding of visual perception in the primate brain
Source: PLoS Comput Biol. 2024 May 6;20(5):e1012058. doi: 10.1371/journal.pcbi.1012058 (PMC11098503; doi:10.1371/journal.pcbi.1012058)
Supplement: S8 Appendix — Fig A: Generating abstract images. Top: abstract image (taken from [34]). Bottom: image corresponding to the iteratively-optimized latent to match its visual features with those of the target latent. (PDF) [file pcbi.1012058.s008.pdf]

S8 Appendix: Abstract Stimuli

Our experiment did not include abstract stimuli or optical illusions, as in [34]. It is however worth noting that the feasibility thereof, using StyleGAN-XL's framework, is an intriguing question. To this end, we (partially) leveraged the inversion script which can be found in the original GitHub repository of StyleGAN-XL. In brief, we optimized an input w-latent via the perceptual LPIPS loss using VGG16 features. We used the default parameters as specified in the script. Our findings suggest that, within the limitations of StyleGAN-XL's design tied to the natural image distribution, the generator indeed exhibits the capability to synthesize such images (Fig A in S8 Appendix). This, in turn, offers an interesting perspective for future investigations in this direction. Note that inverting via the extended W^+ latent space could result in generated images that match the input images even more closely.

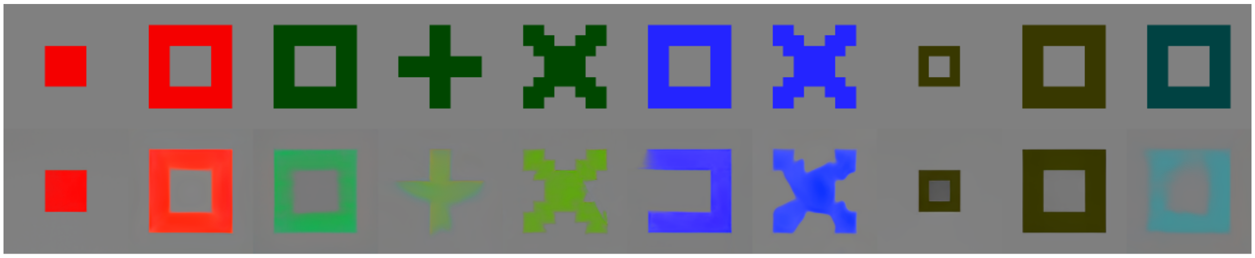

Fig A: **Generating abstract images.** Top: abstract image (taken from [34]). Bottom: image corresponding to the iteratively-optimized latent to match its visual features with those of the target latent.
