# Supplementary material for: Brain2GAN: Feature-disentangled neural encoding and decoding of visual perception in the primate brain
Source: PLoS Comput Biol. 2024 May 6;20(5):e1012058. doi: 10.1371/journal.pcbi.1012058 (PMC11098503; doi:10.1371/journal.pcbi.1012058)
Supplement: S9 Appendix — (PDF) [file pcbi.1012058.s009.pdf]

S9 Appendix: Category labels (Tiny ImageNet [75])

1. Egyptian cat
2. reel
3. volleyball
4. rocking chair
5. lemon
6. bullfrog
7. basketball
8. cliff
9. espresso
10. plunger
11. parking meter
12. German shepherd
13. dining table
14. monarch
15. brown bear
16. school bus
17. pizza
18. guinea pig
19. umbrella
20. organ
21. oboe
22. maypole
23. goldfish
24. potpie
25. hourglass
26. seashore
27. computer keyboard
28. Arabian camel
29. ice cream
30. nail
31. space heater
32. cardigan
33. baboon
34. snail
35. coral reef
36. albatross
37. spider web
38. sea cucumber
39. backpack
40. Labrador retriever
41. pretzel
42. king penguin
43. sulphur butterfly
44. tarantula
45. lesser panda
46. pop bottle
47. banana
48. sock
49. cockroach
50. projectile
51. beer bottle
52. mantis
53. freight car
54. guacamole
55. remote control
56. European fire salamander

57. lakeside
58. chimpanzee
59. pay-phone
60. fur coat
61. alp
62. lampshade
63. torch
64. abacus
65. moving van
66. barrel
67. tabby
68. goose
69. koala
70. bullet train
71. CD player
72. teapot
73. birdhouse
74. gazelle
75. academic gown
76. tractor
77. ladybug
78. miniskirt
79. golden retriever
80. triumphal arch
81. cannon
82. neck brace
83. sombrero
84. gasmask
85. candle
86. desk
87. frying pan
88. bee
89. dam
90. spiny lobster
91. police van
92. iPod
93. punching bag
94. beacon
95. jellyfish
96. wok
97. potter's wheel
98. sandal
99. pill bottle
100. butcher shop
101. slug
102. hog
103. cougar
104. crane
105. vestment
106. dragonfly
107. cash machine
108. mushroom
109. jinrikisha
110. water tower
111. chest
112. snorkel
113. sunglasses
114. fly
115. limousine
116. black stork
117. dugong

118. sports car
119. water jug
120. suspension bridge
121. ox
122. ice lolly
123. turnstile
124. Christmas stocking
125. broom
126. scorpion
127. wooden spoon
128. picket fence
129. rugby ball
130. sewing machine
131. steel arch bridge
132. Persian cat
133. refrigerator
134. barn
135. apron
136. Yorkshire terrier
137. swimming trunks
138. stopwatch
139. lawn mower
140. thatch
141. fountain
142. black widow
143. bikini
144. plate
145. teddy
146. barbershop
147. confectionery
148. beach wagon
149. scoreboard
150. orange
151. flagpole
152. American lobster
153. trolleybus
154. drumstick
155. dumbbell
156. brass
157. bow tie
158. convertible
159. bighorn
160. orangutan
161. American alligator
162. centipede
163. syringe
164. go-kart
165. brain coral
166. sea slug
167. cliff dwelling
168. mashed potato
169. viaduct
170. military uniform
171. pomegranate
172. chain
173. kimono
174. comic book
175. trilobite
176. bison
177. pole
178. boa constrictor

179. poncho
180. bathtub
181. grasshopper
182. walking stick
183. Chihuahua
184. tailed frog
185. lion
186. altar
187. obelisk
188. beaker
189. bell pepper
190. bannister
191. bucket
192. magnetic compass
193. meat loaf
194. gondola
195. standard poodle
196. acorn
197. lifeboat
198. binoculars
199. cauliflower
200. African elephant
